# Mesenchymal stromal cell therapy in liver disease: opportunities and lessons to be learnt?

**DOI:** 10.1152/ajpgi.00036.2015

**Published:** 2015-08-27

**Authors:** Andrew Owen, Philip N. Newsome

**Affiliations:** ^1^National Institute for Health Research Birmingham Liver Biomedical Research Unit and Centre for Liver Research, Birmingham, United Kingdom; and; ^2^Liver Unit, University Hospital Birmingham National Health Service Foundation Trust, Birmingham, United Kingdom

**Keywords:** liver disease, mesenchymal stem cell, mesenchymal stromal cell

## Abstract

End-stage liver disease is responsible for 30,000 deaths per year in the United States alone, and it is continuing to increase every year. With liver transplantation the only curative treatment currently available, new therapies are in great demand. Mesenchymal stem cells (MSC) offer an opportunity to both treat liver inflammatory damage, as well as reverse some of the changes that occur following chronic liver injury. With the ability to regulate both the innate and adaptive immune system, as well as both inhibit and promote apoptosis of effector inflammatory cells, there are numerous therapeutic opportunities for MSC in acute and chronic liver disease. This article critically appraises the potential therapeutic roles of MSC in liver disease, as well as the barriers to their adoption into clinical practice.

in the united states (us) there are ∼30,000 deaths each year due to chronic liver disease, which is increasing at a rate of 3 percent per year (67). Currently, the only curative treatment for end stage liver disease is transplantation, but there are over 15,000 patients on the waiting list for a liver transplant operation in the US, and ∼50% of these patients will never receive a transplant (103a). In the United Kingdom (UK) the problem is similar with 2% of all deaths being due to liver disease, and while all other leading causes of death are decreasing, those from end-stage liver disease have increased by 20% (69, 113). Notably, liver disease is the leading cause of premature death in the UK, leading to the loss of a greater number of life years than many of the other causes. Clearly, novel therapeutic options are needed to reduce the global impact of liver diseases; mesenchymal stem cells (MSC) are one potential therapy that offer great promise.

MSC are multipotent, self-renewing cells of mesodermal origin that have the potential to differentiate down chondrocytic, osteocytic, and adipocytic lineages among many others (78). MSC exist in a number of tissues, albeit in low numbers (87), and have traditionally been isolated because of their ability to adhere to tissue culture plastic and proliferate (28). This review will cover the possible roles of MSC in liver disease along with their potential pitfalls.

## Evolution of a Bone Marrow Derived Stem Cell

The history of MSC and the development of hypotheses regarding their existence date back over 100 years ago; indeed, a description of bone marrow stroma creating an environment in which hematopoietic precursors were able to differentiate was first suggested as far back as 1908 by Maximov (27, 61). Experiments in the 1960s by Tavassoli confirmed the osteogenic potential of bone marrow, but limitations with these experiments meant it was not possible to identify which cellular constituents within the bone marrow were responsible (98). Further work by Friedenstein demonstrated that a rare population of bone marrow cells with fibroblastic properties were responsible, and the term colony-forming unit fibroblast was used to describe them (99). These cells have subsequently been shown to be multipotent (71), but their complex interplay with hematopoietic stem cells has only recently been demonstrated (62). The term MSC was not used until 1991 when it was introduced by Caplan (16), and the idea of a stem cell niche within the bone marrow was further developed by the discovery of a rare, self-renewing population of cells (87), leading to an ongoing debate regarding the correct criteria with which to judge MSC. This is due to their mixture of stem- and stromal cell-like properties, although the ability to self-renew and tri-lineage differentiation potential (osteogenic, chondrogenic, and adipogenic) appear in most definitions (13, 22).

The definition of MSC in humans has focused on their adherence to tissue culture plastic, multipotency, and expression profile of specific cell surface antigens (Table 1). As regard the latter, a population of putative human MSC should be greater than 95% positive for positive antigens and contain less than 2% positivity for negative antigens (22, 39). In mice however, CD105, CD90, and VCAM-1 have been identified as relevant markers for MSC purity (Table 1), although successful isolation of MSC from murine bone marrow has proven challenging (19, 74), leading to the isolation of markedly heterogeneous cell populations and potentially inconsistent results in preclinical studies. Prospective isolation of MSC subpopulations using cell-sorting techniques has been demonstrated in both mice and humans. Highly purified mouse MSC obtained from bone marrow by sorting on PDGFRα and Sca-1 expression (with depletion of cells expressing Ter119 and CD45) demonstrate tri-lineage differentiation and self-renewal (66). In humans and mice, MSC can also be isolated on the basis of their LNGFR^+^ (CD271), THY-1^+^, and VCAM-1^hi+^ (59) expression profile and again have been shown to undergo tri-lineage differentiation and self-renewal. The intermediate filament protein nestin has also been shown to identify a population of perivascular MSC, which are able to support the hematopoietic niche (62) and may also be used as a marker for prospective isolation. Notably, the overlap between PαS and nestin-positive cells is not complete, with the majority of nestin^+^ cells not expressing Sca-1, suggesting some phenotypic differences (77).

**Table 1.** Presence and absence of surface markers required for identification of human and mouse MSC

Positive Surface Antigens		Negative Surface Antigens	
Human	Mouse	Human	Mouse
CD105	CD105	CD79α or CD19	CD45
CD90 (Thy1)	CD90	CD45	Ter119
CD73	VCAM	CD34	
CD71	PDFRα	CD14 or CD11b	
CD44	Sca1	HLA-DR	
GD2		CD86	
LNGFR (CD271)		CD80	
		CD40	

## 

### MSC and immunomodulation.

The number of studies looking at MSC in the laboratory and clinical setting has increased dramatically over the past decade due to their pleiotropic actions in respect to regeneration and immunomodulation. Their immunomodulatory properties apply to both the adaptive and the innate immune systems and are seemingly mediated by a combination of migration to inflamed tissues, as well as by remote signaling (103, 109, 117).

**Fig. 1.**
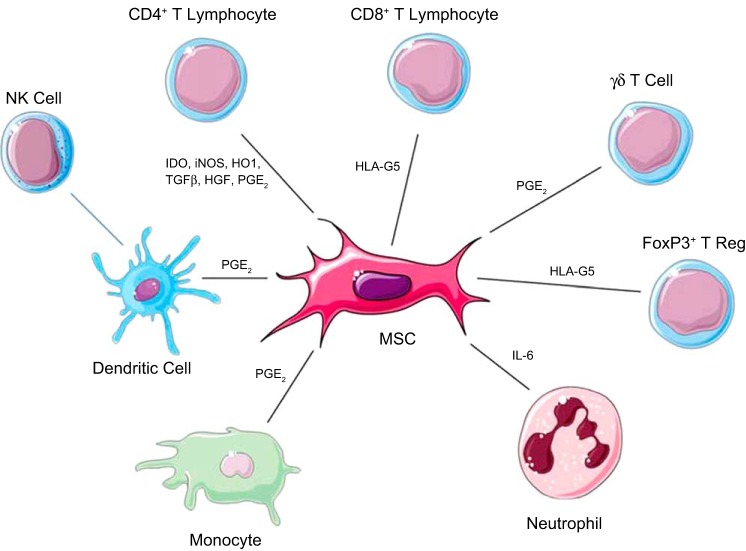
Immune cells influenced by mesenchymal stem cells (MSC). MSC exert an effect on a range of cells involved in the immune response. There is a direct effect exerted on CD4^+^, CD8^+^, γδT-Cells, FoxP3^+^ T-reg cells, neutrophils, and monocytes, while they also exert an indirect effect on natural killer (NK) cells via their action on dendritic cells. (Stock images provided by Servier medical for use under the Creative Commons Attribution 3.0 Unported License).

Regarding the innate immune system MSC can inhibit the maturation of dendritic cells (18, 44, 80), as well as decrease their expression of MHC class 1, MHC class 2, and other costimulatory molecules, thus reducing their antigen-presenting ability. It has been demonstrated in vitro that MSC can inhibit the release of TNF-α by dendritic cells via a PGE_2_-dependent mechanism and also stimulate plasmacytoid dendritic cells to increase their production of IL-10 (1). This reduction in inflammation is one of the mechanisms proposed for the success of MSC in graft vs. host disease (55, 63). MSC also have an inhibitory effect on natural killer (NK) cells likely due to the release of soluble factors such as indoleamine-2,3-dioxygenase (IDO), TGF-β, PGE_2_, and IL-10 (Table 2 and Fig. 1). This inhibitory effect has been shown to prevent activation of NK cells by IL-2; however, once NK cells are activated, the inhibitory effect of MSC is only partial, measured by reductions in IFNγ secretion by NK cells (95). MSC can be induced to increase their production of myosin heavy chain class 1 and 2 by activation using IFNγ, which has been shown to protect MSC from NK-induced apoptosis (95).

**Table 2.** Factors Secreted By MSC Known To Be Important in Immunomodulation

Cytokine	Effect
Nerve growth factor (NGF)	Binds to P75 on hepatic stellate cells and triggers apoptosis
Interleukin 6 (Il-6)	Inhibits neutrophil burst
Inducible nitric oxide synthetase (iNOS)	Inhibits CD4^+^ T-cell function
Indolamine 2,3 dioxygenase (IDO)	Inhibits CD4^+^ T-cell function, inhibits resting natural killer cells
Prostaglandin E_2_ (PGE_2_)	Inhibits CD4^+^ T-cell function, inhibits resting natural killer cells, inhibits differentiation of monocytes into myeloid cells, inhibits TNF production by dendritic cells
Hepatocyte growth factor (HGF)	Inhibits CD4^+^ T-cell function, inhibits CD8^+^ T-cell cytotoxicity
Transforming growth factor β (TGF-β)	Inhibits CD4^+^ T-cell function
Human leucocyte antigen G5 (HLA-G5)	Inhibits resting natural killer cells

Regarding the adaptive immune system, MSC are able to inhibit T-cell proliferation and their activation; however, the precise mechanisms by which this is achieved are unclear. Early cell cycle arrest of T cells may have a role in their suppressive action, and MSC have been shown to inhibit cyclin D2 and upregulate p27Kip1, and although the mechanism is not clear, this process is independent of MHC expression (33). The suppressive activity of MSC is not however, limited to a specific subset of T cells and has also been shown to occur during CD40L and IL-4 stimulation of B cells, which likely reflects the role of cyclin D2 in driving B-cell proliferation (82, 118). Notably, an inflammatory environment is required for MSC to exert their immunosuppressive effect, as otherwise, MSC have been shown paradoxically to exert a proinflammatory effect on T-cells (68).

As well as human and mouse MSC possessing phenotypically different expression profiles, there are also differences between strains of mice and rats with respect to their mode of immunomodulation, with BALBc mice predominantly secreting inducible nitric oxide, as opposed to IDO (9, 35). Human MSC have also been shown to favor IDO as their mechanism of immunosuppression (26); thus, it is critical to choose the correct strain of mice for MSC isolation when carrying out studies with a translational objective.

## MSC in Liver Disease

The role of MSC has been studied in a range of different settings of liver disease, with widely varying actions reported, ranging from reduction of oxidative stress, paracrine trophic signals to hepatocytes, to suppression of immune responses and reduction of liver fibrosis. The majority of the literature regarding MSC usage in patients with liver disease is made up of either observational studies or case series. Table 3 summarizes some of the key controlled trials carried out in this area.

**Table 3.** Key clinical studies in liver disease

	Study Design	Number of Patients Treated	Etiology of Liver Failure	Type of MSC	Route of Administration	Cell Numbers	Predefined Primary Endpoint	Outcome
Acute on Chronic Liver Failure								
Shi et al., *Stem Cells Translational Medicine*, 2012	Open labeled nonrandomized controlled trial	24	Hepatitis B infection	UC MSC	Peripheral intravenous infusion	0.5 × 10^6^·kg^−3^· wk^−1^ at 4-wk intervals for 3 cycles	No	Improved survival at 72 wk (20.8% vs. 47.4% *P* = 0.015)
Peng et al., *Hepatology*, 2011	Nonrandomized controlled trial	57	Hepatitis B infection	Autologous BM MSC	Hepatic artery infusion	3.8 × 10^8^ by single injection	No	Improvement in MELD score at 36 wk (15.55 vs. 18.79)
Fibrosis/Cirrhosis								
Zhang et al., *Journal of Gastroenterology and Hepatology*, 2012	Open labeled nonrandomized controlled trial	30	Chronic Hepatitis B infection	UC MSC	Peripheral intravenous infusion	0.5 × 10^6^/kg every 4 wk on 3 occasions	No	Improvement in ascites volume assessed by ultrasound (5 mm vs. 22 mm at 50 wk) and albumin levels (32 vs. 35 *P* < 0.05)
Salama et al., *Stem Cell Res Therapeutics*, 2014	Randomized controlled trial	20	Hepatitis C infection	Autologous BM MSC	Peripheral intravenous infusion	1.0 × 10^6^/kg by single infusion	No	Global improvement in liver function tests ≤6 mo (bilirubin 2.06 vs. 4.24, INR 1.52 vs. 1.84, ALT fold 1.27 vs. 1.09)
Mohamadnejad et al., *Liver International*, 2013	Randomized controlled trial	15	Mixed	Autologous BM MSC	Peripheral intravenous infusion	1.20–2.95 × 10^8^ by single infusion	No	3 deaths in MSC treated group. No significant difference in MELD score or liver function tests
Xu et al., *Journal of Gastroenterology and Hepatology*, 2014	Randomized controlled trial	20	Hepatitis B infection	Autologous BM MSC	Hepatic artery infusion	8.45 × 10^8^ by single infusion	No	Improvement in MELD score (11 vs. 9) and ALT (30 vs. 25) at 24 wk

MSC, mesenchymal stem cells; UC, umbilical cord; BM, bone marrrow; MELD, Model for End-Stage Liver Disease; ALT, alanine aminotransferase.

### Acute liver failure/acute on chronic liver failure.

The rapid onset of liver failure in patients without preexisting liver disease is relatively uncommon but has considerable morbidity and mortality, despite improvements in critical care provision and liver transplant surgery (35a). Causes of acute liver failure vary between the developing world, where viral infection is the major cause, and the developed world, where drug-induced liver injury is more common (12). Drug-induced liver injury is responsible for 50% of cases of acute liver failure in the United States (84) and Europe, where the main drug responsible is acetaminophen (11).

In preclinical models of acute liver damage, such as from carbon tetrachloride (CCl_4_) and concanavalin A, MSC have been shown to reduce liver injury (46, 120), although the mechanisms by which this is achieved are complex and not fully understood. A reduction in proinflammatory cytokines, in particular, TNFα, IFNγ, and IL-4, may be responsible and in some studies appears to be greater with repeated dosing of MSC (17). In parallel with the reduction in inflammatory cytokines, a reduction in hepatocyte apoptosis has also been demonstrated using the TUNEL assay (48). Oxidative stress plays a role in a number of liver injury models, including CCL_4_-induced liver injury and hepatic ischemia reperfusion. In CCL_4_-induced liver injury, MSC have been shown to reduce oxidative stress, with parallel in vitro experiments, demonstrating an ability to act as a free radical scavenger, reducing the amount of reactive oxygen species available (53). In acetaminophen-induced liver injury, damage can be reduced by inhibition of JNK (34), which MSC have been demonstrated to achieve along with reductions in hepatic JNK and TNF-α and maintenance of levels of hepatic glutathione (89).

MSC-conditioned media (MSC-CM) and MSC based extracorporeal membranes (MSC-EM) appear to be as effective, if not more so, than MSC infusions alone in certain models. In d-galactosamine-induced liver injury models, MSC-EM appear to show a greater reduction in hepatocyte death and reversal of fulminant hepatic failure, followed by MSC-CM with cellular infusion showing the lowest effect (73). However, MSC-CM contains over 50 cytokines, and it is yet to be elucidated what combination of these is most effective in the treatment of liver disease. The inability to, thus, identify a defined product is likely to be problematic for regulators when trying to translate this finding into clinical practice.

In the developing world, viral hepatitis is the most common cause of acute liver failure, mainly due to hepatitis A (49, 93) and E (39), although hepatitis B has also been shown to cause acute on chronic liver failure (91). Early clinical trials in this latter setting have shown the potential benefit of MSC therapy (119), with a reduction in ascites volume, as well as improvements in liver function and serum albumin level, although these studies were not formal clinical trials with identified primary end-points and, thus, need confirmation in future studies. The mechanisms by which MSC may exert their beneficial effects in this setting are also not clear and requires study (94). Of note, hepatitis B virus is able to infect MSC, although the implications of this are not entirely clear (58).

## Ischemia Reperfusion and Transplantation

**Fig. 2.**
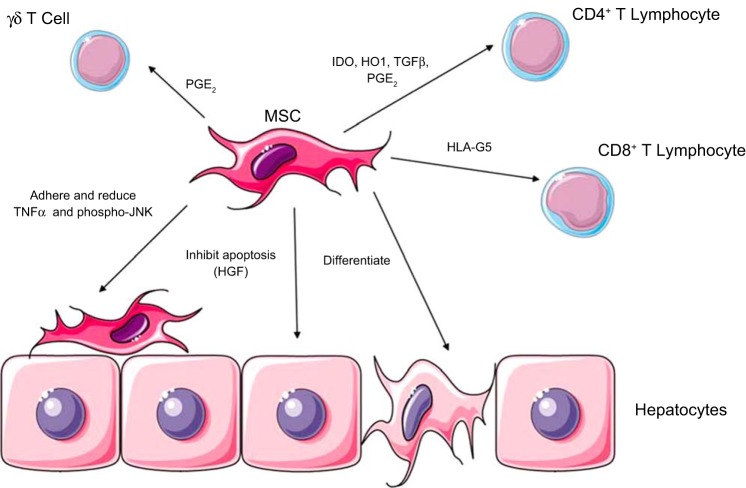
Mechanisms of MSC action in liver inflammation/ischemia. MSC are able to inhibit CD4, CD8, and γδ T lymphocytes using a variety of cytokines, including LHA-G5, IDO, HO1, TGFβ, and PGE_2_. MSC may also differentiate into hepatocytes, although this occurs in low numbers. Hepatocyte apoptosis is inhibited by MSC, secreting HGF, and finally MSC may adhere to hepatocytes and reduce TNF-α and phosphor-JNK. (Stock images provided by Servier medical for use under the Creative Commons Attribution 3.0 Unported License).

Ischemic liver injury is an underrecognized clinical condition occurring at its most dramatic during the ischemia-reperfusion injury accompanying liver transplantation (54), as well as during episodes of hypoperfusion, such as cardiac arrest, trauma, and sepsis (14). Recruitment of CD4^+^ and CD8^+^ T cells coupled with natural killer (NK) and γδT cells occurs early in ischemia reperfusion (IR) injury and is a key pathogenic mechanism in the development of the immunity-mediated liver injury (105) seen in this setting. In preclinical studies, inhibition of leukocyte adhesion significantly reduces liver injury in the setting of transplant-induced ischemia-reperfusion injury (100). Hence, MSC, which have a potent ability to suppress T-cell activity and proliferation, have been proposed as therapeutic adjuncts in this setting (Fig. 2).

Hepatocyte transplantation has been carried out in both acute liver failure (96) and inborn errors of metabolism (21), with mixed results. Failure of sufficient engraftment and rejection of transplanted hepatocytes restrict the clinical utility of this approach, and thus, an adjunctive role of MSC has been proposed. Notably, MSC have been shown to prolong hepatocyte survival both in vitro and in vivo, as well as maintain their function (45, 104). MSC can also downregulate the number of TUNEL-positive hepatocytes in partial hepatic injury models, as well as increasing the number of proliferating hepatocytes (104), and, thus, may prolong hepatocyte retention after transplantation. MSC have also been shown in vitro to differentiate into hepatocytes (6, 70) and/or fuse with hepatocytes adopting their phenotype (101). While initially thought to be a mechanism by which MSC are able to support tissue repair and regeneration, the low numbers of such cells suggests that this function of MSC is not the most important feature, with inhibition of apoptosis a more likely explanation.

In preclinical studies of reduced-size liver transplantation, MSC have been show to provide trophic support for donor livers and improve recipient survival, although the exact mechanisms were not studied (23). Notably, infusions of MSC that had been transfected with an HGF adenovirus vector to stimulate their production of HGF have been shown to further improve survival in the setting of small for-size liver transplantation, although the relative contributions of HGF and/or MSC in this setting require further study (115, 116). MSC have also been shown to significantly reduce AST and ALT, as well as decrease the number of apoptotic hepatocytes, as assessed by the TUNEL assay in a model of hepatic IR (48). As the MSC in this study adhered to the peri-portal region and appeared to have reduced the number of apoptotic hepatocytes, it seems likely that a paracrine effect is responsible for this, and reductions in TNF-α and phospho-JNK by MSC are possible mechanisms.

Tolerogenic properties of MSC have been investigated in a rat model of liver transplantation. Following orthotopic liver transplantation, MSC infusion has been shown to increase tolerance to donor organs by suppressing T-cell levels, as well as increasing the number of circulating CD4^+^CD25^+^FoxP3^+^ regulatory T cells (108, 111). Clinical translation of this effect has yet to be demonstrated; however, although studies in kidney transplantation look encouraging (76, 83, 97). Neutrophils are a key component of the inflammatory insult seen following ischemia-reperfusion injury. MSC have been shown to exert an effect on neutrophils. With reciprocal modulation of the mitochondrial proteins of the Bcl2 family; Bax and MCL-1, MSC can inhibit neutrophil apoptosis, even at a low ratio (1:500) (79). In coculture experiments, MSC have been shown to increase the amount of phosphorylated STAT3 via secretion of IL-6, a likely explanation for their ability to inhibit neutrophil apoptosis.

## Chronic Liver Disease

**Fig. 3.**
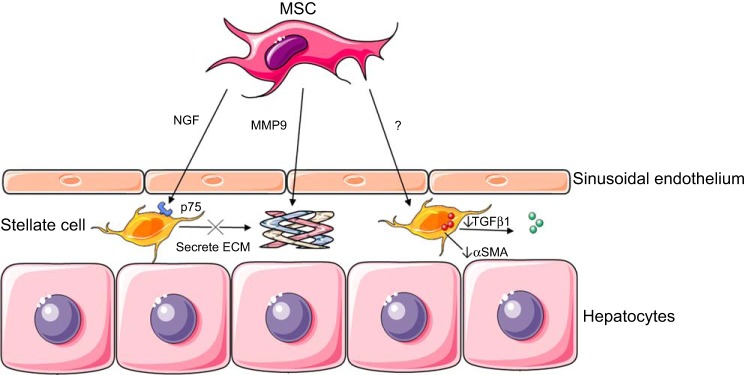
Mechanisms of MSC action in fibrotic liver disease. MSC can exert effects on hepatic stellate cells by secreting nerve growth factor (NGF), which binds to p75 expressed on activated stellate cells. This leads to stellate cell apoptosis and, therefore, a reduction in the stellate cell secreted ECM. MSC may also secrete MMP9, which has a direct effect of cleaving collagen in the ECM. MSC also act via an unknown mechanism to reduce the secretion of stellate cell αSMA and TGFβ. (Stock images provided by Servier medical for use under the Creative Commons Attribution 3.0 Unported License).

Liver fibrosis is the common final result of most chronic liver diseases, and while once thought of as an irreversible phenomenon, there is optimism that it may be amenable to specific antifibrotic therapies (29). Cirrhosis, the most severe manifestation of liver fibrosis, represents the consequence of stellate cell activation following chronic liver injury and the deposition of extracellular matrix (ECM) proteins and collagen (30). Fibrosis can coexist with ongoing inflammatory injury and, thus, MSC have been explored as both anti-inflammatory and antifibrotic therapies in this setting. Activation of stellate cells contributes to fibrogenesis, and MSC-induced initiation of stellate cell apoptosis has been suggested as a potential treatment for hepatic fibrosis (72) (Fig. 3).

In preclinical trials, MSC have been shown to improve liver function in models of cirrhosis and decrease expression of α-SMA, TGF-β1, and type 1 collagen (47). Activated stellate cells express the receptor P75, which triggers apoptosis in response to nerve growth factor (NGF) probably by induction of the C-Jun N-terminal kinase and NF-κB pathways. MSC may increase stellate cell apoptosis via the release of NGF (57). Matrix metalloproteinase 9 (MMP9) is a protease known to break down the ECM, and MSC have been shown to increase the expression of MMP9, along the fibrous septa in mouse models, leading to regression of fibrosis (37). As an explanation for their regenerative effect in liver fibrosis, it has been proposed that bone marrow-derived stem cells may migrate to injured liver and differentiate into hepatocytes (70); however, alternative mechanisms seem more likely. A key criticism of the preclinical work carried out using MSC in liver fibrosis/cirrhosis is that MSC were often administered at or during the injury period, and thus, there is uncertainty as to whether they are having an anti-inflammatory or antifibrotic effect. With little mechanistic insight provided by the current preclinical work, further study focusing on the way in which MSC may exert their effects in these models is required.

One clinical trial in patients with liver cirrhosis demonstrated an increased liver volume in patients treated with MSC (51); however, this was an observational study, and a subsequent randomized trial showed no beneficial effects of MSC therapy in this patient group (65). A recent review of trials using MSC in end-stage liver disease has demonstrated the paucity of good quality trials with very few randomized controlled trials in these patients (60). There is a need for good quality open-label randomized trials to be carried out without predefined end points to better answer the question of the benefit of MSC in end-stage liver disease.

In clinical trials, it has also been suggested that MSC therapy may improve liver function in patients with end-stage liver disease due to hepatitis C virus, as supported by a downregulation in fibrosis markers and proinflammatory cytokines (88), alongside an upregulation of anti-inflammatory cytokines. MSC have also been shown to improve liver function in patients with cirrhosis secondary to chronic hepatitis B infection (75), with significant improvements in liver function tests when compared with antivirals alone, possibly by increasing the number of Treg (FoxP3^+^) cells and decreasing the number of Th17 (IL-17 T-helper) cells (114), hence altering the Treg/Th17 ratio. Moreover, some studies suggest that the MSC secretome may be as effective as MSC themselves, raising questions about their mechanism of action and possibly negating the risk of stem cell infusion (7). Clearly there is considerable further work to explore the mechanisms by which MSC may be beneficial in fibrotic liver disease with immunity-mediated interactions being the key focus of investigation.

## Delivery of Cellular Therapy and Engraftment at Target Sites

A number of routes of administration have been proposed for the delivery of MSC in liver disease. While the classic routes of intravenous infusion or subcutaneous injection are more familiar, other routes such as intra-arterial and intraperitoneal injection, either via ultrasound guidance or at time of surgery have also been investigated (50). The proposed advantage of the intraperitoneal route over other routes in liver disease is the ability to circumvent the lungs, an area in which a large proportion of intravenous MSC will ultimately accumulate (8). Choice of route will also be determined by a balance between what is clinically practical, hence, the preference for systemic administration.

Homing and engraftment are important concepts when considering delivery of MSC to specific target organs such as the liver. We have demonstrated that MSC use CD29 and CD44 to mediate adhesion to sinusoidal endothelium in the CCl_4_ mouse model of liver injury, thus increasing engraftment (4). Although human MSC have also been shown to express CCR7, CCR9, CXCR4, CXCR5, and CXCR6 (38), receptors that are involved in homeostatic leukocyte tracking, it remains unclear whether these are important in homing to injured tissue. Stimulating MSC to upregulate receptors used for engraftment may be one strategy to increase and target MSC homing. MSC cultured under standard conditions quickly lose CXCR4 expression; however, when cultured under hypoxic conditions CXCR4 expression is increased and may aid homing to tissues expressing SDF-1α, such as bone marrow and ischemic tissues (41). Cytokine stimulation in hematopoietic stem cells has been shown to increase expression of receptors responsible for engraftment and may represent another potential strategy in MSC therapy.

While the importance of homing/engraftment of MSC to the injured liver is assumed to be necessary, recent work in liver fibrosis and other clinical settings such as graft vs. host disease has questioned this premise. Encapsulated MSC (eMSC) demonstrated a superior effect when compared with intravenously administered MSC, which may be a reflection of prolonged survival of the eMSC in vivo (117). This work suggests that MSC may exert their effects through paracrine signaling or cell contact with circulating inflammatory cells, such as myeloid derived stromal or dendritic cells (18).

Techniques enabling the tracking of MSC will be important so as to better understand their in vivo action. One early technique for tracking cell distribution was magnetic resonance imaging (MRI), of supermagnetic iron oxide (SPIO)-labeled cells (107). There is evidence, however, that the labeling process itself, magnetoporation, can inhibit MSC differentiation and migration, limiting the usefulness of this technology (90). One alternative to SPIOs are manganese oxide nanoparticles, which have been used to track MSC in mouse models of glioblastoma (40). Radionucleotide reporter gene imaging using single photon emission computed tomography is a potential alternative to MRI; however, it is limited by the spillover of radiation to nonlabeled cell types and the short time frame with which imaging is possible because of radionucleotide decay (43).

## Clinical Translation of MSC Therapy

Although MSC have potentially broad-reaching clinical applications in liver disease they have yet to demonstrate unequivocal evidence of efficacy. This predominantly reflects the lack of robust phase 2/3 clinical trials performed with the rigor required by regulators to meet predefined primary end-points.

As a rare population of cells, MSC requires extensive culture expansion to yield enough cell numbers for a clinical effect, which raises concern about loss of function (31) and potentially transformation. Undeniably overexpansion of MSC in culture reduces their ability to immunosuppress subsequently, and, thus, regimens defining maximal expansion before use are required, along with robust release assays, which are predictive of in vivo functionality. Concerns about transformation of MSC in culture pertain only to murine studies (3), whereas human studies do not suggest any evidence of transformation. Indeed, initial evidence of oncogenesis was retracted, as it was later shown to be due to cell line contamination (32). Longer-term studies of patients receiving human MSC also demonstrated the lack of any long-term engraftment, providing further reassurance on this matter (106).

Of note, MSC require exposure to inflammation to induce their immunomodulatory actions, whereas in a quiescent environment, they may adopt a proinflammatory phenotype (5, 112). The desire, therefore, to prime MSC to enhance their function poses a dilemma between additional logistical and financial challenges at the expense of potentially greater efficacy.

Inflammatory bowel disease is another group of conditions caused by immune dysregulation in which MSC may have potential benefit; however, so far, only small case series have been performed. One study has demonstrated an improvement in mucosal inflammation (56), and another study demonstrated improvement in half of the patients and deterioration in the others (24) following administration of MSC.

### Tumorigenicity.

Tumor promotion has been considered as a potential risk with MSC therapy, as theoretically immunosuppression could serve to encourage tumor initiation, and MSC secrete angiogenic factors such as VEGF and PDGF which may serve to promote tumor growth (10). The possibility that MSC can give rise to tumor-associated fibroblasts has also been considered in the literature (64), although MSC therapy in the setting of hepatocellular carcinoma has been shown to both inhibit tumor growth via downregulation of Wnt signaling pathway associated factors, while promoting tumor growth by secretion of trophic factors in other models (36). The heterogeneity in the literature is possibly a reflection of transformation of MSC, which is found more commonly in the murine setting, especially after isolation using plastic adherence techniques (52).

Two reports of spontaneous transformation of human MSC upon transplantation (85, 86) led to the suspension of several human plastic adherent (PA)-MSC clinical trials, although both reports (85, 86) were subsequently retracted, as rigorous analysis revealed that the PA-MSC used in both studies was cross-contaminated by the human HT1080 fibrosarcoma cancer cell line (20, 102). To date, only one report has ever demonstrated that human adult tissue-derived PA-MSC can spontaneously transform. Wang et al. (110) generated PA-MSC lines from over 100 donors, and of these lines, one donor PA-MSC line formed tumors in nonobese diabetic severe combined immunodeficiency (NOD/SCID) immunity-compromised mice (110). This cell displayed an abnormal (non-International Society for Cellular Therapy) cell surface cytometry profile of CD133^+^, CD90^low^, CD105^−^, VEGFR2^+^, whereas normal PA-MSC express high levels of CD90^+^ and CD105^+^, but do not express CD133 or VEGFR2 in culture. Karyotyping showed chromosome aneuploidy, and these cells expressed a high level of telomerase activity, compared with typical PA-MSC. As a result of this study, every clinical grade batch of PA-MSC currently undergoes karyotyping and flow cytometry as batch release criteria.

More robust data using rodent PA-MSC have raised concerns that the use of rodent MSC can lead to cancer in certain rodent models either directly or through promotion of existing early stage cancer. Miura et al. (64a) showed that murine MSC could bypass senescence and passage 65 MSC injected into mice formed fibrosarcomas in multiple organs. Raising additional concerns, Breitbach et al. (15) reported that murine PA-MSC led to ectopic bone formation in infarcted mouse hearts. Foudah et al. (25) report that rat MSC (rMSC) exhibited genomic instability and tumorigenicity in culture, leading to the conclusion that rat MSC may not be a good model for exploring the therapeutic potential of human MSC. Jeong et al. (42) extended these findings, showing that murine MSC exhibit genetic instabilities at low passages and lead to tumors in the heart and hindlimbs of mice. Chromosomal analysis revealed that culturing these normal-looking, tumorigenic mouse PA-MSC cause multiple chromosomal abnormalities. These reports must be taken seriously, however, as the increased susceptibility of inbred rodent cells to transformation is well described, these findings may not be surprising, and when considering human cells, it should be noted that they are reportedly resistant to oncogenesis. The Weinberg lab has described that tumorigenic transformation of normal human fibroblasts requires the mutation of six different signaling pathways, whereas mouse fibroblasts require only two pathway mutations (p53 and Raf) to bypass senescence and transform (81).

Nonetheless, careful monitoring for adverse effects of PA-MSC therapies in nonclinical and clinical settings continues to support an acceptable safety profile for PA-MSC with regard to proliferation or ectopic tissue formation. Finally, recent autopsy data from GvHD clinical trial patients that received PA-MSC between 2002 and 2007, revealed no ectopic tissue, neoplasms, or donor-derived DNA (106).

### Conclusions.

MSC have been shown to reduce immunity-mediated liver injury, oxidative stress, and stimulate liver regeneration in a range of preclinical models, but there still remains a lack of detailed studies delineating the mechanisms by which MSC achieve their effects. While the clinical translation of these effects is yet to be confirmed in large-scale randomized trials, there are an increasing number of such studies under way. These studies will hopefully shed light on which clinical indications are appropriate, as well as provide added insights on dosing regimen and safety profile.

## GRANTS

A.O. was funded by an MRC Clinical Research Fellowship. P. N. Newsome is supported by NIHR. This paper presents independent work funded by the National Institute for Health Research (NIHR). The views expressed are those of the authors(s) and not necessarily those of the NHS, the NIHR, or the Department of Health.

## DISCLOSURES

No conflicts of interest, financial or otherwise, are declared by the author(s).

## AUTHOR CONTRIBUTIONS

A.O. prepared figures; A.O. and P.N.N. drafted manuscript; A.O. and P.N.N. edited and revised manuscript; A.O. and P.N.N. approved final version of manuscript.

## References

[1] Aggarwal S, Pittenger MF Human mesenchymal stem cells modulate allogeneic immune cell responses. Blood 105: 1815–1822, 2005. 10.1182/blood-2004-04-1559 15494428

[3] Aguilar S, Nye E, Chan J, Loebinger M, Spencer-Dene B, Fisk N, Stamp G, Bonnet D, Janes SM Murine but not human mesenchymal stem cells generate osteosarcoma-like lesions in the lung. Stem Cells 25: 1586–1594, 2007. 17363552 10.1634/stemcells.2006-0762

[4] Aldridge V, Garg A, Davies N, Bartlett DC, Youster J, Beard H, Kavanagh DP, Kalia N, Frampton J, Lalor PF, Newsome PN Human mesenchymal stem cells are recruited to injured liver in a β_1_-integrin and CD44 dependent manner. Hepatology 56: 1063–1073, 2012. 22422467 10.1002/hep.25716

[5] Anton K, Banerjee D, Glod J Macrophage-associated mesenchymal stem cells assume an activated, migratory, pro-inflammatory phenotype with increased IL-6 and CXCL10 secretion. PLoS One 7: e35036, 2012. 22496888 10.1371/journal.pone.0035036 PMC3319627

[6] Aurich I, Mueller LP, Aurich H, Luetzkendorf J, Tisljar K, Dollinger MM, Schormann W, Walldorf J, Hengstler JG, Fleig WE, Christ B Functional integration of hepatocytes derived from human mesenchymal stem cells into mouse livers. Gut 56: 405–415, 2007. 16928726 10.1136/gut.2005.090050 PMC1856821

[7] Bai L, Lennon DP, Caplan AI, DeChant A, Hecker J, Kranso J, Zaremba A, Miller RH Hepatocyte growth factor mediates mesenchymal stem cell-induced recovery in multiple sclerosis models. Nat Neurosci 15: 862–870, 2012. 22610068 10.1038/nn.3109 PMC3427471

[8] Barbash IM, Chouraqui P, Baron J, Feinberg MS, Etzion S, Tessone A, Miller L, Guetta E, Zipori D, Kedes LH, Kloner RA, Leor J Systemic delivery of bone marrow-derived mesenchymal stem cells to the infarcted myocardium: feasibility, cell migration, and body distribution. Circulation 108: 863–868, 2003. 12900340 10.1161/01.CIR.0000084828.50310.6A

[9] Barzilay R, Sadan O, Melamed E, Offen D Comparative characterization of bone marrow-derived mesenchymal stromal cells from four different rat strains. Cytotherapy 11: 435–442, 2009. 10.1080/14653240902849796 19521891

[10] Beckermann BM, Kallifatidis G, Groth A, Frommhold D, Apel A, Mattern J, Salnikov AV, Moldenhauer G, Wagner W, Diehlmann A, Saffrich R, Schubert M, Ho AD, Giese N, Buchler MW, Friess H, Buchler P, Herr I VEGF expression by mesenchymal stem cells contributes to angiogenesis in pancreatic carcinoma. Br J Cancer 99: 622–631, 2008. 18665180 10.1038/sj.bjc.6604508 PMC2527820

[11] Bernal W, Auzinger G, Dhawan A, Wendon J Acute liver failure. Lancet 376: 190–201, 2010. 20638564 10.1016/S0140-6736(10)60274-7

[12] Bernal W, Wendon J Acute liver failure. N Engl J Med 369: 2525–2534, 2013. 10.1056/NEJMra1208937 24369077

[13] Bianco P, Cao X, Frenette PS, Mao JJ, Robey PG, Simmons PJ, Wang CY The meaning, the sense and the significance: translating the science of mesenchymal stem cells into medicine. Nat Med 19: 35–42, 2013. 10.1038/nm.3028 PMC3998103 23296015

[14] Birrer R, Takuda Y, Takara T Hypoxic hepatopathy: pathophysiology and prognosis. Int Med 46: 1063–1070, 2007. 10.2169/internalmedicine.46.0059 17634701

[15] Breitbach M, Bostani T, Roell W, Xia Y, Dewald O, Nygren JM, Fries JW, Tiemann K, Bohlen H, Hescheler J, Welz A, Bloch W, Jacobsen SE, Fleischmann BK Potential risks of bone marrow cell transplantation into infarcted hearts. Blood 110: 1362–1369, 2007. 17483296 10.1182/blood-2006-12-063412

[16] Caplan A Mesenchymal stem cells. J Orthop Res 9: 641–650, 1991. 1870029 10.1002/jor.1100090504

[17] Chen Y, Chen S, Liu LY, Zou ZL, Cai YJ, Wang JG, Chen B, Xu LM, Lin Z, Wang XD, Chen YP Mesenchymal stem cells ameliorate experimental autoimmune hepatitis by activation of the programmed death 1 pathway. Immunol Lett 162: 222–228, 2014. 25445618 10.1016/j.imlet.2014.10.021

[18] Chiesa S, Morbelli S, Morando S, Massollo M, Marini C, Bertoni A, Frassoni F, Bartolome ST, Sambuceti G, Traggiai E, Uccelli A Mesenchymal stem cells impair in vivo T-cell priming by dendritic cells. Proc Natl Acad Sci USA 108: 17,384–17,389, 2011. 10.1073/pnas.1103650108 PMC3198360 21960443

[19] da Silva Meirelles L, Chagastelles P, and Nardi N Mesenchymal stem cells reside in virtually all post-natal organs and tissues. J Cell Sci 119: 2204–2213, 2006. 16684817 10.1242/jcs.02932

[20] de la Fuente R, Bernad A, Garcia-Castro J, Martin MC, Cigudosa JC Retraction: Spontaneous human adult stem cell transformation. Cancer Res 70: 6682, 2010. 20710046 10.1158/0008-5472.CAN-10-2451

[21] Dhawan A, Mitry RR, Hughes RD Hepatocyte transplantation for liver-based metabolic disorders. J Inherit Metab Dis 29: 431–435, 2006. 16763914 10.1007/s10545-006-0245-8

[22] Dominici M, Le Blank K, Mueller I, Slaper-Cortenbach I, Marini FC, Krause DS, Deans RJ, Keating A, Prockop DJ, Horwitz EM Minimal criteria for defining multipotent mesenchymal stromal cells. The International Society for Cellular Therapy position statement. Cytotherapy 8: 315–317, 2006. 16923606 10.1080/14653240600855905

[23] Du Z, Wei C, Cheng K, Han B, Yan J, Zhang M, Peng C, Liu Y Mesenchymal stem cell-conditioned medium reduces liver injury and enhances regeneration in reduced-size rat liver transplantation. J Surg Res 183: 907–915, 2013. 23522455 10.1016/j.jss.2013.02.009

[24] Duijvestein M, Vos AC, Roelofs H, Wildenberg ME, Wendrich BB, Verspaget HW, Kooy-Winkelaar EM, Koning F, Zwaginga JJ, Fidder HH, Verhaar AP, Fibbe WE, van den Brink GR, Hommes DW Autologous bone marrow-derived mesenchymal stromal cell treatment for refractory luminal Crohn's disease: results of a phase I study. Gut 59: 1662–1669, 2010. 20921206 10.1136/gut.2010.215152

[25] Foudah D, Redaelli S, Donzelli E, Bentivegna A, Miloso M, Dalpra L, Tredici G Monitoring the genomic stability of in vitro cultured rat bone-marrow-derived mesenchymal stem cells. Chromosome Res 17: 1025–1039, 2009. 19957104 10.1007/s10577-009-9090-6 PMC2793379

[26] Francois M, Romieu-Mourez R, Li M, Galipeau J Human MSC suppression correlates with cytokine induction of indoleamine 2,3-dioxygenase and bystander M2 macrophage differentiation. Mol Ther 20: 187–195, 2012. 21934657 10.1038/mt.2011.189 PMC13064680

[27] Friedenstein A Stromal-hematopoietic interrelationships: Maximov's ideas and modern models. Haematol Blood Transfus 32: 159–167, 1989. 2696677 10.1007/978-3-642-74621-5_27

[28] Friedenstein AJ, Chailakhjan RK, Lalykina KS The development of fibroblast colonies in monolayer cultures of guinea-pig bone marrow and spleen cells. Cell Tissue Kinet 3: 393–403, 1970. 5523063 10.1111/j.1365-2184.1970.tb00347.x

[29] Friedman SL Liver fibrosis—from bench to bedside. J Hepatol 38 Suppl 1: S38–S53, 2003. 12591185 10.1016/s0168-8278(02)00429-4

[30] Friedman SL Mechanisms of disease: mechanisms of hepatic fibrosis and therapeutic implications. Nat Clin Pract Gastroenterol Hepatol 1: 98–105, 2004. 16265071 10.1038/ncpgasthep0055

[31] Galipeau J Concerns arising from MSC retrieval from cryostorage and effect on immune suppressive function and pharmaceutical usage in clinical trials. ISBT Sci Ser 8: 100–101, 2013.

[32] Garcia S, Bernad A, Martin MC, Cigudosa JC, Garcia-Castro J, de la Fuente R Pitfalls in spontaneous in vitro transformation of human mesenchymal stem cells. Exp Cell Res 316: 1648–1650, 2010. 20171963 10.1016/j.yexcr.2010.02.016

[33] Glennie S, Soeiro I, Dyson PJ, Lam EW, Dazzi F Bone marrow mesenchymal stem cells induce division arrest anergy of activated T cells. Blood 105: 2821–2827, 2005. 15591115 10.1182/blood-2004-09-3696

[34] Gunawan BK, Liu ZX, Han D, Hanawa N, Gaarde WA, Kaplowitz N c-Jun N-terminal kinase plays a major role in murine acetaminophen hepatotoxicity. Gastroenterology 131: 165–178, 2006. 16831600 10.1053/j.gastro.2006.03.045

[35] Hashemi SM, Hassan ZM, Pourfathollah AA, Soudi S, Shafiee A, Soleimani M Comparative immunomodulatory properties of adipose-derived mesenchymal stem cells conditioned media from BALB/c, C57BL/6, and DBA mouse strains. J Cell Biochem 114: 955–965, 2013. 23225199 10.1002/jcb.24437

[35a] **Health Resources and Services Administration**. 2007 Annual Report of the U.S. Organ Procurement and Transplantation Network and the Scientific Registry of Transplant Recipients (OPTN/SRTR): Transplant Data 1997–2006. In: Annual Report of the U.S. Organ Procurement and Transplantation Network and the Scientific Registry of Transplant Recipients. Rockville, MD: Health Resources and Services Administration, Healthcare Systems Bureau, Division of Transplantation, 2007.

[36] Hernanda PY, Pedroza-Gonzalez A, van der Laan LJ, Broker ME, Hoogduijn MJ, Ijzermans JN, Bruno MJ, Janssen HL, Peppelenbosch MP, Pan Q Tumor promotion through the mesenchymal stem cell compartment in human hepatocellular carcinoma. Carcinogenesis 34: 2330–2340, 2013. 23740837 10.1093/carcin/bgt210 PMC3786382

[37] Higashiyama R, Inagaki Y, Hong YY, Kushida M, Nakao S, Niioka M, Watanabe T, Okano H, Matsuzaki Y, Shiota G, Okazaki I Bone marrow-derived cells express matrix metalloproteinases and contribute to regression of liver fibrosis in mice. Hepatology 45: 213–222, 2007. 17187438 10.1002/hep.21477

[38] Honczarenko M, Le Y, Swierkowski M, Ghiran I, Glodek AM, Silberstein LE Human bone marrow stromal cells express a distinct set of biologically functional chemokine receptors. Stem Cells 24: 1030–1041, 2006. 16253981 10.1634/stemcells.2005-0319

[39] Hoofnagle J, Nelson K, Purcell ER Hepatitis. N Engl J Med 367: 1237–1244, 2012. 23013075 10.1056/NEJMra1204512

[40] Huang J, Xie J, Chen K, Bu L, Lee S, Cheng Z, Li X, Chen X HSA coated MnO nanoparticles with prominent MRI contrast for tumor imaging. Chem Commun 46: 6684–6686, 2010. 10.1039/c0cc01041c PMC3629962 20730157

[41] Hung SC, Pochampally RR, Hsu SC, Sanchez C, Chen SC, Spees J, Prockop DJ Short-term exposure of multipotent stromal cells to low oxygen increases their expression of CX3CR1 and CXCR4 and their engraftment in vivo. PLoS One 2: e416, 2007. 17476338 10.1371/journal.pone.0000416 PMC1855077

[42] Jeong JO, Han JW, Kim JM, Cho HJ, Park C, Lee N, Kim DW, Yoon YS Malignant tumor formation after transplantation of short-term cultured bone marrow mesenchymal stem cells in experimental myocardial infarction and diabetic neuropathy. Circ Res 108: 1340–1347, 2011. 21493893 10.1161/CIRCRESAHA.110.239848 PMC3109741

[43] Jiang H, Cheng Z, Tian M, Zhang H In vivo imaging of embryonic stem cell therapy. Eur J Nucl Med Mol Imag 38: 774–784, 2011. 10.1007/s00259-010-1667-y 21107558

[44] Jiang XX, Zhang Y, Liu B, Zhang SX, Wu Y, Yu XD, Mao N Human mesenchymal stem cells inhibit differentiation and function of monocyte-derived dendritic cells. Blood 105: 4120–4126, 2005. 15692068 10.1182/blood-2004-02-0586

[45] Joshi M, PBP, He z, Holgersson J, Olausson M, Sumitran-Holgersson S Fetal liver-derived mesenchymal stromal cells augment engraftment of transplanted hepatocytes. Cytotherapy 14: 657–669, 2012. 22424216 10.3109/14653249.2012.663526 PMC3411318

[46] Jung J, Choi JH, Lee Y, Park JW, Oh IH, Hwang SG, Kim KS, Kim GJ Human placenta-derived mesenchymal stem cells promote hepatic regeneration in CCl_4_-injured rat liver model via increased autophagic mechanism. Stem Cells 31: 1584–1596, 2013. 23592412 10.1002/stem.1396

[47] Jung KH, Shin HP, Lee S, Lim YJ, Hwang SH, Han H, Park HK, Chung JH, Yim SV Effect of human umbilical cord blood-derived mesenchymal stem cells in a cirrhotic rat model. Liver Int 29: 898–909, 2009. 19422480 10.1111/j.1478-3231.2009.02031.x

[48] Kanazawa H, Fujimoto Y, Teratani T, Iwasaki J, Kasahara N, Negishi K, Tsuruyama T, Uemoto S, Kobayashi E Bone marrow-derived mesenchymal stem cells ameliorate hepatic ischemia reperfusion injury in a rat model. PLoS One 6: e19195, 2011. 21559442 10.1371/journal.pone.0019195 PMC3084802

[49] Kar P, Budhiraja S, Narang A, Chakravarthy A Etiology of sporadic acute and fulminant non-A, non-B viral hepatitis in north India. Indian J Gastroenterol 16: 43–45, 1997. 9114568

[50] Kharaziha P, Hellstrom PM, Noorinayer B, Farzaneh F, Aghajani K, Jafari F, Telkabadi M, Atashi A, Honardoost M, Zali MR, Soleimani M Improvement of liver function in liver cirrhosis patients after autologous mesenchymal stem cell injection: a phase I–II clinical trial. Eur J Gastroenterol Hepatol 21: 1199–1205, 2009. 10.1097/MEG.0b013e32832a1f6c 19455046

[51] Kim JK, Park YN, Kim JS, Park MS, Paik YH, Seok JY, Chung YE, Kim HO, Kim KS, Ahn SH, Kim do Y, Kim MJ, Lee KS, Chon CY, Kim SJ, Terai S, Sakaida I, Han KH Autologous bone marrow infusion activates the progenitor cell compartment in patients with advanced liver cirrhosis. Cell Transplant 19: 1237–1246, 2010. 20525430 10.3727/096368910X506863

[52] Klopp AH, Gupta A, Spaeth E, Andreeff M, Marini F 3rd Dissecting a discrepancy in the literature: do mesenchymal stem cells support or suppress tumor growth? Stem Cells 29: 11–19, 2011. 21280155 10.1002/stem.559 PMC3059412

[53] Kuo TK, Hung SP, Chuang CH, Chen CT, Shih YRV, Fang SCY, Yang VW, Lee OK Stem cell therapy for liver disease: parameters governing the success of using bone marrow mesenchymal stem cells. Gastroenterology 134: 2111–2121. e2113, 2008. 18455168 10.1053/j.gastro.2008.03.015 PMC3086672

[54] Kupiec-Weglinski JW, Busuttil RW Ischemia and reperfusion injury in liver transplantation. Transplant Proc 37: 1653–1656, 2005. 15919422 10.1016/j.transproceed.2005.03.134

[55] Le Blanc K, Frassoni F, Ball L, Locatelli F, Roelofs H, Lewis I, Lanino E, Sundberg B, Bernardo ME, Remberger M, Dini G, Egeler RM, Bacigalupo A, Fibbe W, Ringden O Mesenchymal stem cells for treatment of steroid-resistant, severe, acute graft-versus-host disease: a phase II study. Lancet 371: 1579–1586, 2008. 18468541 10.1016/S0140-6736(08)60690-X

[56] Liang J, Zhang H, Wang D, Feng X, Wang H, Hua B, Liu B, Sun L Allogeneic mesenchymal stem cell transplantation in seven patients with refractory inflammatory bowel disease. Gut 61: 468–469, 2012. 21617158 10.1136/gutjnl-2011-300083

[57] Lin N, Hu K, Chen S, Xie S, Tang Z, Lin J, Xu R Nerve growth factor-mediated paracrine regulation of hepatic stellate cells by multipotent mesenchymal stromal cells. Life Sci 85: 291–295, 2009. 19559033 10.1016/j.lfs.2009.06.007

[58] Ma R, Xing Q, Shao L, Wang D, Hao Q, Li X, Sai L, Ma L Hepatitis B virus infection and replication in human bone marrow mesenchymal stem cells. Virol J 8: 486, 2011. 22035170 10.1186/1743-422X-8-486 PMC3225454

[59] Mabuchi Y, Morikawa S, Harada S, Niibe K, Suzuki S, Renault-Mihara F, Houlihan Diarmaid D, Akazawa C, Okano H, Matsuzaki Y LNGFR+THY-1+VCAM-1hi+ cells reveal functionally distinct subpopulations in mesenchymal stem cells. Stem Cell Reports 1: 152–165, 2013. 24052950 10.1016/j.stemcr.2013.06.001 PMC3757748

[60] Margini C, Vukotic R, Brodosi L, Bernardi M, Andreone P Bone marrow derived stem cells for the treatment of end-stage liver disease. World J Gastroenterol 20: 9098–9105, 2014. 25083082 10.3748/wjg.v20.i27.9098 PMC4112892

[61] Maximov AA Uber experimentelle Erzeugung von Knochenmarks-Gewebe [in German]. Anat Anz 28: 24–38, 1906.

[62] Méndez-Ferrer S, Michurina TV, Ferraro F, Mazloom AR, MacArthur BD, Lira SA, Scadden DT, Ma'ayan A, Enikolopov GN, Frenette PS Mesenchymal and haematopoietic stem cells form a unique bone marrow niche. Nature 466: 829–834, 2010. 20703299 10.1038/nature09262 PMC3146551

[63] Min CK, Kim BG, Park G, Cho B, Oh IH IL-10-transduced bone marrow mesenchymal stem cells can attenuate the severity of acute graft-versus-host disease after experimental allogeneic stem cell transplantation. Bone Marrow Transplant 39: 637–645, 2007. 17369865 10.1038/sj.bmt.1705644

[64] Mishra PJ, Mishra PJ, Humeniuk R, Medina DJ, Alexe G, Mesirov JP, Ganesan S, Glod JW, Banerjee D Carcinoma-associated fibroblast-like differentiation of human mesenchymal stem cells. Cancer Res 68: 4331–4339, 2008. 18519693 10.1158/0008-5472.CAN-08-0943 PMC2725025

[64a] Miura M, Miura Y, Padilla-Nash HM, Molinolo AA, Fu B, Patel V, Seo BM, Sonoyama W, Zheng JJ, Baker CC, Chen W, Ried T, Shi S Accumulated chromosomal instability in murine bone marrow mesenchymal stem cells leads to malignant transformation. Stem Cells 24: 1095–1103, 2006. 16282438 10.1634/stemcells.2005-0403

[65] Mohamadnejad M, Alimoghaddam K, Bagheri M, Ashrafi M, Abdollahzadeh L, Akhlaghpoor S, Bashtar M, Ghavamzadeh A, Malekzadeh R Randomized placebo-controlled trial of mesenchymal stem cell transplantation in decompensated cirrhosis. Liver Int 33: 1490–1496, 2013. 23763455 10.1111/liv.12228

[66] Morikawa S, Mabuchi Y, Kubota Y, Nagai Y, Niibe K, Hiratsu E, Suzuki S, Miyauchi-Hara C, Nagoshi N, Sunabori T, Shimmura S, Miyawaki A, Nakagawa T, Suda T, Okano H, Matsuzaki Y Prospective identification, isolation, and systemic transplantation of multipotent mesenchymal stem cells in murine bone marrow. J Exp Med 206: 2483–2496, 2009. 19841085 10.1084/jem.20091046 PMC2768869

[67] Murphy SL, Xu J, Kochanek KD, Division of Vital Statistics. Deaths: Final Data for 2010, U.S. Department of Health and Human Services. Natl Vital Stat Rep 61: 1–118, 2013. 24979972

[68] Najar M, Rouas R, Raicevic G, Boufker HI, Lewalle P, Meuleman N, Bron D, Toungouz M, Martiat P, Lagneaux L Mesenchymal stromal cells promote or suppress the proliferation of T lymphocytes from cord blood and peripheral blood: the importance of low cell ratio and role of interleukin-6. Cytotherapy 11: 570–583, 2009. 19565371 10.1080/14653240903079377

[69] Office of National Statistics. U.K. Health of the Population Causes of Death, 2014, Newport, UK: Office of National Statistics, 2015.

[70] Oh SH, Witek RP, Bae SH, Zheng D, Jung Y, Piscaglia AC, Petersen BE Bone marrow-derived hepatic oval cells differentiate into hepatocytes in 2-acetylaminofluorene/partial hepatectomy-induced liver regeneration. Gastroenterology 132: 1077–1087, 2007. 17383429 10.1053/j.gastro.2007.01.001

[71] Owen M, Friedenstein A Stromal stem cells: marrow-derived osteogenic precursors. Ciba Found Symp 136: 42–60, 1988. 3068016 10.1002/9780470513637.ch4

[72] Parekkadan B, van Poll D, Megeed Z, Kobayashi N, Tilles AW, Berthiaume F, Yarmush ML Immunomodulation of activated hepatic stellate cells by mesenchymal stem cells. Biochem Biophys Res Commun 363: 247–252, 2007. 17869217 10.1016/j.bbrc.2007.05.150 PMC2096777

[73] Parekkadan B, van Poll D, Suganuma K, Carter EA, Berthiaume F, Tilles AW, Yarmush ML Mesenchymal stem cell-derived molecules reverse fulminant hepatic failure. PLoS One 2: e941, 2007. 17895982 10.1371/journal.pone.0000941 PMC1978513

[74] Peister A, Mellad J, Larson B, Hall B, Gibson L, Prockop D Adult stem cells from bone marrow (MSCs) isolated from different strains of inbred mice vary in surface epitopes, rates of proliferation, and differentiation potential. Blood 103: 1662–1668, 2004. 14592819 10.1182/blood-2003-09-3070

[75] Peng L, Xie DY, Lin BL, Liu J, Zhu HP, Xie C, Zheng YB, Gao ZL Autologous bone marrow mesenchymal stem cell transplantation in liver failure patients caused by hepatitis B: short-term and long-term outcomes. Hepatology 54: 820–828, 2011. 21608000 10.1002/hep.24434

[76] Perico N, Casiraghi F, Gotti E, Introna M, Todeschini M, Cavinato RA, Capelli C, Rambaldi A, Cassis P, Rizzo P, Cortinovis M, Noris M, Remuzzi G Mesenchymal stromal cells and kidney transplantation: pretransplant infusion protects from graft dysfunction while fostering immunoregulation. Transplant Int 26: 867–878, 2013. 10.1111/tri.12132 23738760

[77] Pinho S, Lacombe J, Hanoun M, Mizoguchi T, Bruns I, Kunisaki Y, Frenette PS PDGFR and CD51 mark human Nestin+ sphere-forming mesenchymal stem cells capable of hematopoietic progenitor cell expansion. J Exp Med 210: 1351–1367, 2013. 23776077 10.1084/jem.20122252 PMC3698522

[78] Pittenger MF, Mackay AM, Beck SC, Jaiswal RK, Douglas R, Mosca JD, Moorman MA, Simonetti DW, Craig S, Marshak DR Multilineage potential of adult human mesenchymal stem cells. Science 284: 143–147, 1999. 10102814 10.1126/science.284.5411.143

[79] Raffaghello L, Bianchi G, Bertolotto M, Montecucco F, Busca A, Dallegri F, Ottonello L, Pistoia V Human mesenchymal stem cells inhibit neutrophil apoptosis: a model for neutrophil preservation in the bone marrow niche. Stem Cells 26: 151–162, 2008. 17932421 10.1634/stemcells.2007-0416

[80] Ramasamy R, Fazekasova H, Lam EW, Soeiro I, Lombardi G, Dazzi F Mesenchymal stem cells inhibit dendritic cell differentiation and function by preventing entry into the cell cycle. Transplantation 83: 71–76, 2007. 17220794 10.1097/01.tp.0000244572.24780.54

[81] Rangarajan A, Weinberg RA Comparative biology of mouse versus human cells: modelling human cancer in mice. Nat Rev Cancer 3: 952–959, 2003. 14737125 10.1038/nrc1235

[82] Rasmusson I, Ringden O, Sundberg B, Le Blanc K Mesenchymal stem cells inhibit lymphocyte proliferation by mitogens and alloantigens by different mechanisms. Exp Cell Res 305: 33–41, 2005. 15777785 10.1016/j.yexcr.2004.12.013

[83] Reinders ME, de Fijter JW, Roelofs H, Bajema IM, de Vries DK, Schaapherder AF, Claas FH, van Miert PP, Roelen DL, van Kooten C, Fibbe WE, Rabelink TJ Autologous bone marrow-derived mesenchymal stromal cells for the treatment of allograft rejection after renal transplantation: results of a phase I study. Stem Cells Transl Med 2: 107–111, 2013. 23349326 10.5966/sctm.2012-0114 PMC3659754

[84] Reuben A, Koch DG, Lee WM Drug-induced acute liver failure: results of a U.S. multicenter, prospective study. Hepatology 52: 2065–2076, 2010. 20949552 10.1002/hep.23937 PMC3992250

[85] Rosland GV, Svendsen A, Torsvik A, Sobala E, McCormack E, Immervoll H, Mysliwietz J, Tonn JC, Goldbrunner R, Lonning PE, Bjerkvig R, Schichor C Long-term cultures of bone marrow-derived human mesenchymal stem cells frequently undergo spontaneous malignant transformation. Cancer Res 69: 5331–5339, 2009. 19509230 10.1158/0008-5472.CAN-08-4630

[86] Rubio D, Garcia-Castro J, Martin MC, de la Fuente R, Cigudosa JC, Lloyd AC, Bernad A Spontaneous human adult stem cell transformation. Cancer Res 65: 3035–3039, 2005. 15833829 10.1158/0008-5472.CAN-04-4194

[87] Sacchetti B, Funari A, Michienzi S, Di Cesare S, Piersanti S, Saggio I, Tagliafico E, Ferrari S, Robey PG, Riminucci M, Bianco P Self-renewing osteoprogenitors in bone marrow sinusoids can organize a hematopoietic microenvironment. Cell 131: 324–336, 2007. 17956733 10.1016/j.cell.2007.08.025

[88] Salama H, Zekri AR, Medhat E, Al Alim SA, Ahmed OS, Bahnassy AA, Lotfy MM, Ahmed R, Musa S Peripheral vein infusion of autologous mesenchymal stem cells in Egyptian HCV-positive patients with end-stage liver disease. Stem Cell Res Ther 5: 70, 2014. 24886681 10.1186/scrt459 PMC4097846

[89] Salomone F, Barbagallo I, Puzzo L, Piazza C, Li Volti G Efficacy of adipose tissue-mesenchymal stem cell transplantation in rats with acetaminophen liver injury. Stem Cell Res 11: 1037–1044, 2013. 23954692 10.1016/j.scr.2013.07.003

[90] Schafer R, Kehlbach R, Muller M, Bantleon R, Kluba T, Ayturan M, Siegel G, Wolburg H, Northoff H, Dietz K, Claussen CD, Wiskirchen J Labeling of human mesenchymal stromal cells with superparamagnetic iron oxide leads to a decrease in migration capacity and colony formation ability. Cytotherapy 11: 68–78, 2009. 19191056 10.1080/14653240802666043

[91] Schiodt FV, Atillasoy E, Shakil AO, Schiff ER, Caldwell C, Kowdley KV, Stribling R, Crippin JS, Flamm S, Somberg KA, Rosen H, McCashland TM, Hay JE, Lee WM Etiology and outcome for 295 patients with acute liver failure in the United States. Liver Transplant Surg 5: 29–34, 1999. 10.1002/lt.500050102 9873089

[93] Shah U, Habib Z, Kleinman RE Liver failure attributable to hepatitis A virus infection in a developing country. Pediatrics 105: 436–438, 2000. 10654972 10.1542/peds.105.2.436

[94] Shi M, Zhang Z, Xu R, Lin H, Fu J, Zou Z, Zhang A, Shi J, Chen L, Lv S, He W, Geng H, Jin L, Liu Z, Wang FS Human mesenchymal stem cell transfusion is safe and improves liver function in acute-on-chronic liver failure patients. Stem Cells Transl Med 1: 725–731, 2012. 23197664 10.5966/sctm.2012-0034 PMC3659658

[95] Spaggiari GM Mesenchymal stem cell-natural killer cell interactions: evidence that activated NK cells are capable of killing MSCs, whereas MSCs can inhibit IL-2-induced NK-cell proliferation. Blood 107: 1484–1490, 2006. 16239427 10.1182/blood-2005-07-2775

[96] Strom SC, Chowdhury JR, Fox IJ Hepatocyte transplantation for the treatment of human disease. Sem Liver Dis 19: 39–48, 1999. 10.1055/s-2007-1007096 10349682

[97] Tan J, Wu W, Xu X, Liao L, Zheng F, Messinger S, Sun X, Chen J, Yang S, Cai J, Gao X, Pileggi A, Ricordi C Induction therapy with autologous mesenchymal stem cells in living-related kidney transplants: a randomized controlled trial. JAMA 307: 1169–1177, 2012. 22436957 10.1001/jama.2012.316

[98] Tavassoli M, Crosby W Transplantation of marrow to extramedullary sites. Science 161: 54–56, 1968. 4871792 10.1126/science.161.3836.54

[99] Tavassoli M, Friedenstein A Hemopoietic stromal microenvironment. Am J Hematol 15: 195–203, 1989. 6613987 10.1002/ajh.2830150211

[100] Teoh NC, Ito Y, Field J, Bethea NW, Amr D, McCuskey MK, McCuskey RS, Farrell GC, Allison AC Diannexin, a novel annexin V homodimer, provides prolonged protection against hepatic ischemia-reperfusion injury in mice. Gastroenterology 133: 632–646, 2007. 17681182 10.1053/j.gastro.2007.05.027

[101] Terada N, Hamazaki T, Oka M, Hoki M, Mastalerz DM, Nakano Y, Meyer EM, Morel L, Petersen BE, Scott EW Bone marrow cells adopt the phenotype of other cells by spontaneous cell fusion. Nature 416: 542–545, 2002. 11932747 10.1038/nature730

[102] Torsvik A, Rosland GV, Svendsen A, Molven A, Immervoll H, McCormack E, Lonning PE, Primon M, Sobala E, Tonn JC, Goldbrunner R, Schichor C, Mysliwietz J, Lah TT, Motaln H, Knappskog S, Bjerkvig R Spontaneous malignant transformation of human mesenchymal stem cells reflects cross-contamination: putting the research field on track. Cancer Res 70: 6393–6396, 2010. 20631079 10.1158/0008-5472.CAN-10-1305

[103] Uccelli A, Moretta L, Pistoia V Mesenchymal stem cells in health and disease. Nat Rev Immunol 8: 726–736, 2008. 19172693 10.1038/nri2395

[103a] **U.S. Department of Health and Human Services**. Organ Procurement and Transplantation Network, 2014, http://optn.transplant.hrsa.gov/.

[104] van Poll D, Parekkadan B, Cho CH, Berthiaume F, Nahmias Y, Tilles AW, Yarmush ML Mesenchymal stem cell-derived molecules directly modulate hepatocellular death and regeneration in vitro and in vivo. Hepatology 47: 1634–1643, 2008. 18395843 10.1002/hep.22236

[105] Vardanian AJ, Busuttil RW, Kupiec-Weglinski JW Molecular mediators of liver ischemia and reperfusion injury: a brief review. Mol Med 14: 337–345, 2008. 18292799 10.2119/2007-00134.Vardanian PMC2247470

[106] von Bahr L, Batsis I, Moll G, Hagg M, Szakos A, Sundberg B, Uzunel M, Ringden O, Le Blanc K Analysis of tissues following mesenchymal stromal cell therapy in humans indicates limited long-term engraftment and no ectopic tissue formation. Stem Cells 30: 1575–1578, 2012. 22553154 10.1002/stem.1118

[107] Walczak P, Kedziorek DA, Gilad AA, Lin S, Bulte JW Instant MR labeling of stem cells using magnetoelectroporation. Magn Reson Med 54: 769–774, 2005. 16161115 10.1002/mrm.20701

[108] Wan CD, Cheng R, Wang HB, Liu T Immunomodulatory effects of mesenchymal stem cells derived from adipose tissues in a rat orthotopic liver transplantation model. Hepatobil Pancreatic Dis Int 7: 29–33, 2008. 18234635

[109] Wang Y, Chen X, Cao W, Shi Y Plasticity of mesenchymal stem cells in immunomodulation: pathological and therapeutic implications. Nat Immunol 15: 1009–1016, 2014. 10.1038/ni.3002 25329189

[110] Wang Y, Huso DL, Harrington J, Kellner J, Jeong DK, Turney J, McNiece IK Outgrowth of a transformed cell population derived from normal human BM mesenchymal stem cell culture. Cytotherapy 7: 509–519, 2005. 10.1080/14653240500363216 16306013

[111] Wang Y, Zhang A, Ye Z, Xie H, Zheng S Bone marrow-derived mesenchymal stem cells inhibit acute rejection of rat liver allografts in association with regulatory T-cell expansion. Transplant Proc 41: 4352–4356, 2009. 20005397 10.1016/j.transproceed.2009.08.072

[112] Waterman RS, Tomchuck SL, Henkle SL, Betancourt AM A new mesenchymal stem cell (MSC) paradigm: polarization into a pro-inflammatory MSC1 or an immunosuppressive MSC2 phenotype. PLoS One 5: e10088, 2010. 20436665 10.1371/journal.pone.0010088 PMC2859930

[113] Williams R, Aspinall R, Bellis M, Camps-Walsh G, Cramp M, Dhawan A, Ferguson J, Forton D, Foster G, Gilmore I, Hickman M, Hudson M, Kelly D, Langford A, Lombard M, Longworth L, Martin N, Moriarty K, Newsome P, O'Grady J, Pryke R, Rutter H, Ryder S, Sheron N, Smith T Addressing liver disease in the UK: a blueprint for attaining excellence in health care and reducing premature mortality from lifestyle issues of excess consumption of alcohol, obesity, and viral hepatitis. Lancet 384: 1953–1997, 2014. 25433429 10.1016/S0140-6736(14)61838-9

[114] Xu L, Gong Y, Wang B, Shi K, Hou Y, Wang L, Lin Z, Han Y, Lu L, Chen D, Lin X, Zeng Q, Feng W, Chen Y Randomized trial of autologous bone marrow mesenchymal stem cells transplantation for hepatitis B virus cirrhosis: regulation of Treg/Th17 cells. J Gastroenterol Hepatol 29: 1620–1628, 2014. 24942592 10.1111/jgh.12653

[115] Yu Y, Lu L, Qian X, Chen N, Yao A, Pu L, Zhang F, Li X, Kong L, Sun B, Wang X Antifibrotic effect of hepatocyte growth factor-expressing mesenchymal stem cells in small-for-size liver transplant rats. Stem Cells Dev 19: 903–914, 2010. 20025519 10.1089/scd.2009.0254

[116] Yu Y, Yao AH, Chen N, Pu LY, Fan Y, Lv L, Sun BC, Li GQ, Wang XH Mesenchymal stem cells over-expressing hepatocyte growth factor improve small-for-size liver grafts regeneration. Mol Ther 15: 1382–1389, 2007. 17519892 10.1038/sj.mt.6300202

[117] Zanotti L, Sarukhan A, Dander E, Castor M, Cibella J, Soldani C, Trovato AE, Ploia C, Luca G, Calvitti M, Mancuso F, Arato I, Golemac M, Jonjic N, Biondi A, Calafiore R, Locati M, D'Amico G, Viola A Encapsulated mesenchymal stem cells for in vivo immunomodulation. Leukemia 27: 500–503, 2012. 22878604 10.1038/leu.2012.202

[118] Zappia E, Casazza S, Pedemonte E, Benvenuto F, Bonanni I, Gerdoni E, Giunti D, Ceravolo A, Cazzanti F, Frassoni F, Mancardi G, Uccelli A Mesenchymal stem cells ameliorate experimental autoimmune encephalomyelitis inducing T-cell anergy. Blood 106: 1755–1761, 2005. 15905186 10.1182/blood-2005-04-1496

[119] Zhang Z, Lin H, Shi M, Xu R, Fu J, Lv J, Chen L, Lv S, Li Y, Yu S, Geng H, Jin L, Lau GK, Wang FS Human umbilical cord mesenchymal stem cells improve liver function and ascites in decompensated liver cirrhosis patients. J Gastroenterol Hepatol 2: 112–120, 2012. 22320928 10.1111/j.1440-1746.2011.07024.x

[120] Zhu X, He B, Zhou X, Ren J Effects of transplanted bone-marrow-derived mesenchymal stem cells in animal models of acute hepatitis. Cell Tissue Res 351: 477–486, 2013. 23143676 10.1007/s00441-012-1524-3

